# Anti-neuroinflammatory Activity of *Elephantopus scaber* L. via Activation of Nrf2/HO-1 Signaling and Inhibition of p38 MAPK Pathway in LPS-Induced Microglia BV-2 Cells

**DOI:** 10.3389/fphar.2017.00397

**Published:** 2017-06-21

**Authors:** Chim-Kei Chan, Loh Teng-Hern Tan, Shathiswaran N. Andy, Muhamad Noor Alfarizal Kamarudin, Bey-Hing Goh, Habsah Abdul Kadir

**Affiliations:** ^1^Biomolecular Research Group, Biochemistry Program, Institute of Biological Sciences, Faculty of Science, University of MalayaKuala Lumpur, Malaysia; ^2^Novel Bacteria and Drug Discovery Research Group, School of Pharmacy, Monash University MalaysiaSubang Jaya, Malaysia; ^3^Center of Health Outcomes Research and Therapeutic Safety (Cohorts), School of Pharmaceutical Sciences, University of PhayaoPhayao, Thailand

**Keywords:** *Elephantopus scaber* L., antioxidant, anti-inflammatory, Nrf2, NF-κB

## Abstract

*Elephantopus scaber* L. (family: Asteraceae) has been traditionally utilized as a folkloric medicine and scientifically shown to exhibit anti-inflammatory activities in various *in vivo* inflammatory models. Given the lack of study on the effect of *E. scaber* in neuroinflammation, this study aimed to investigate the anti-neuroinflammatory effect and the underlying mechanisms of ethyl acetate fraction from the leaves of *E. scaber* (ESEAF) on the release of pro-inflammatory mediators in lipopolysaccharide (LPS)-induced microglia cells (BV-2). Present findings showed that ESEAF markedly attenuated the translocation of NF-κB to nucleus concomitantly with the significant mitigation on the LPS-induced production of NO, iNOS, COX-2, PGE_2_, IL-1β, and TNF-α. These inflammatory responses were reduced via the inhibition of p38. Besides, ESEAF was shown to possess antioxidant activities evident by the DPPH and SOD scavenging activities. The intracellular catalase enzyme activity was enhanced by ESEAF in the LPS-stimulated BV-2 cells. Furthermore, the formation of ROS induced by LPS in BV-2 cells was reduced upon the exposure to ESEAF. Intriguingly, the reduction of ROS was found in concerted with the activation of Nrf2 and HO-1. It is conceivable that the activation promotes the scavenging power of antioxidant enzymes as well as to ameliorate the inflammatory response in LPS-stimulated BV-2 cells. Finally, the safety profile analysis through oral administration of ESEAF at 2000 mg/kg did not result in any mortalities, adverse effects nor histopathologic abnormalities of organs in mice. Taken altogether, the cumulative findings suggested that ESEAF holds the potential to develop as nutraceutical for the intervention of neuroinflammatory disorders.

## Introduction

Inflammation as one of the defense responses to eliminate deleterious agents or injured tissues in order to maintain the homeostasis, prevents continued tissue damage and restores normal function (Medzhitov, 2008). Microglia, the resident immune cells of central nervous system, become activated upon the initiation of inflammatory responses that induces detrimental neurotoxic effects by producing excess pro-inflammatory mediators such as prostaglandin E_2_ (PGE_2_), nitric oxide (NO), cytokines, and reactive oxygen species (ROS) (Amor et al., 2010; Fischer and Maier, 2015). However, when activated microglia undergo a prolonged inflammation triggered by a plethora of stimuli could induce neuronal damage to neighboring neurons and leading to various neurodegenerative diseases including multiple sclerosis, Alzheimer’s disease (AD), and Parkinson’s disease (Block and Hong, 2005). Thus, suppression of the inflammatory mediators by activated microglia could provide a therapeutic target for neuroinflammatory diseases.

Mounting evidences have suggested that NF-κB and p38 MAPK signaling transductions contribute to the onset of microglia activation. Upon the stimulation of lipopolysaccharide (LPS), NF-κB translocate to the nucleus further transcribing several pro-inflammatory genes including inducible nitric oxide synthase (iNOS) and cyclooxygenase-2 (COX-2) (Surh et al., 2001; Ghosh and Karin, 2002). Once activated, NF-κB exacerbated and amplified the inflammatory responses by increasing pro-inflammatory mediators including iNOS, COX-2, tumor necrosis factor-α (TNF-α), and interleukin-1β (IL-1β). In addition, p38 MAPK, an upstream mediator, has been implicated in the release of inflammatory signaling molecules. Activation of NF-κB is tightly orchestrated by phosphorylated p38 and further enhanced the production of iNOS and COX-2 (Ajizian et al., 1999; Kefaloyianni et al., 2006). Therefore, targeting NF-κB and p38 has become another therapeutic approach to rescue the neurons from death as well to mitigate the progression of neurodegenerative diseases.

Oxidative stress which is manifested by the aberrant production of ROS and failure of antioxidant defense system (Limón-Pacheco and Gonsebatt, 2009), has been associated with various physiopathological conditions including cancer, aging, and neurodegenerative diseases such as AD and stroke (Kang and Hamasaki, 2005; Mariani et al., 2005). Cognitive deficits associated with AD and aging are due to the increased susceptibility to oxidative stress and inflammation (Rogers et al., 1996; McGeer and McGeer, 2003; Joseph et al., 2005). Recently, it is well documented that nuclear factor-2 erythroid related factor-2 (Nrf2) and heme-oxygenase-1 (HO-1) proteins are recognized as prominent signaling mediators in antioxidant defense mechanism regulating inflammation to prevent neuronal death caused by microgliosis. Upon oxidative stress stimuli, Nrf2 which dissociates from Keap1 translocates to the nucleus and transactivates antioxidant response element (ARE)-encoding genes including superoxide dismutase (SOD), catalase, GSH, and HO-1 (Johnson et al., 2008; Nguyen et al., 2009; Hsieh and Yang, 2013). In addition, the induction of HO-1 via Nrf2 ameliorates the inflammatory response by inhibiting the translocation of NF-κB and production of NO and PGE_2_ (Foresti et al., 2013). As given, there is strong scientific evidence that neuroinflammation plays a crucial role in the pathogenesis of neurodegenerative diseases. Unfortunately, the currently available clinical drugs tested for anti-neuroinflammatory therapy only offer valuable symptomatic relief, but they are often associated with significant and intolerable adverse effects (Nissen and Wolski, 2007; Della-Morte et al., 2014). Therefore, the endeavor of developing effective therapies targeted to the attenuation of neuroinflammation is highly required because the need to develop drugs with low toxicity and good selectivity to central nervous system has never been more convincing (Craft et al., 2005).

In recent years, there has been renewed interest across all areas of medicine in various natural products especially the plant-based medicines previously described in the domain of ethnopharmacology (Goh and Kadir, 2011; Wong et al., 2012; Moghadamtousi et al., 2014). Traditionally, plants have been used to treat various diseases in many developing countries and become the basis of many traditional medicine systems to provide mankind with new remedies (Fabricant and Farnsworth, 2001; Tan et al., 2016a,b; Tang et al., 2016). Since ancient time, phytochemicals have been used to treat inflammation (Tan et al., 2015; Chan et al., 2016; Yong et al., 2016), beginning with the discovery of aspirin as the first anti-inflammatory and analgesic drug (Vane and Botting, 1987). *Elephantopus scaber*, which is known as elephant’s foot, has a multitude of health benefits including wound-healing, fever, dysuria, hepatoprotective, anticancer, antidiabetic, and anti-inflammatory activities (Singh et al., 2005; Daisy et al., 2009; Ho et al., 2009, 2012; Chan et al., 2015). Traditionally, *E. scaber* was used by the Malay midwives as an oxytocin during childbirth and to prevent post-partum inflammation in Malaysia (Ho et al., 2009). It was also applied for the treatment of various inflammatory conditions in several countries including Taiwan, Malaysia, and Indonesia (Tsai and Lin, 1998; Ho et al., 2009). Scientific evidence showed that extracts of *E. scaber* possessed anti-inflammatory activities in various inflammatory agent-induced animal models, including the inhibition of carrageenan- and adjuvant-induced paw edema in rats (Tsai and Lin, 1998), carbon tetrachloride- or LPS-induced acute and chronic liver dysfunction (Lin et al., 1995; Rajesh and Latha, 2001; Hung et al., 2011). However, the anti-neuroinflammatory effect of *E. scaber* on the LPS-induced inflammation in neuronal immune cells and the underlying mechanisms are still elusive. Therefore, in the present study, the antioxidant and anti-neuroinflammatory effect of the ethyl acetate fraction of *E. scaber* leaf and the underlying mechanisms involved were investigated in the LPS-stimulated BV-2 microglia cells.

## Materials and Methods

### Animals and Experimental Design

The animals (C57BL/6 mice), male (25 ± 2 g), 8–10 weeks old were obtained from the Laboratory Animal Centre of the School of Medicine and Health Sciences, Monash University Malaysia. The mice were housed in standard conditions and supplied with standard pelleted feed and water *ad libitum*. Experiment was conducted according to the protocol approved by the Animal Ethics Committee from Monash University (Ref. No. MARP/2014/022).

### Acute Oral Toxicity Assay

Ten mice were assigned into two groups, each groups had five mice. One group is for control and another group is for the treatment. Acute oral toxicity of the ethyl acetate of *E. scaber* leaves (ESEAF) was performed according to the OECD guideline No. 425. For the control group, mice were given sesame oil only. A limit test was performed; one C57BL6 male mice was dosed at 2,000 mg/kg ESEAF dissolved in sesame oil. Four additional mice were sequentially dosed at approximately 48–72 h intervals after the mice was survived. The mice were fasted for 4 h prior to administration and after 3 h of treatment food supply was regained. On the first day of treatment, mortality and signs of toxicity were monitored at 30 min, 1, 3, and 4 h and followed by twice for daily until day 14. Body weights of the mice were documented on day 0 (initiation), 7 and 14 (termination) (Sayyad et al., 2016). After 14 days of oral administration, the control and treated group of mice were anesthetized with Zoletil/Ketamine/Xylazine at the dosage of 13.5 mg/kg. The animals will be sacrificed by cardiac puncture and the blood was collected for biochemical analysis. The organs including brain, kidney, lung, heart, spleen, and liver were excised and weighed.

### Histopathological Examination

The collected organs were fixed in 10% buffered formalin. The tissues were embedded in paraffin wax, sectioned into 4 μm thick tissue and deparaffinized in xylene following by a series of hydration process. The tissues were subjected to hematoxylin and eosin (H&E) staining and further observed under the microscope.

### Blood Biochemical Analysis

Blood serum which obtained from mice were evaluated for the biochemical parameters. Parameters including albumin, total bilirubin levels (TBIL), alanine aminotransferase (ALT), alkaline phosphatase (ALP), aspartate aminotransferase (AST), and creatinine (CRE) were tested.

### Preparation and Extraction of *E. scaber* Leaves

According to our previous study (Chan et al., 2015), the leaves of *E. scaber* (**Figure 2A**) were purchased from a local supplier in June, 2014 and upon authentication, a voucher specimen (No. KLU47976) was deposited at the herbarium in the Institute of Biological Sciences, University of Malaya, Kuala Lumpur, Malaysia. In brief, dried leaves of *E. scaber* was grounded and soaked with 70% ethanol for 3 days. Subsequently, the crude ethanol extract was filtered and further concentrated by rotary evaporator (Buchi). The crude ethanol extract was further fractioned by using hexane. The hexane insoluble fraction was proceeded to solvent-solvent partition using ethyl acetate and water (Lau et al., 2015). The ethyl acetate-soluble fraction was evaporated to obtain *E. scaber* leaves’ ethyl acetate fraction (ESEAF) which later was dissolved in DMSO prior to each assay. The final concentration of DMSO was maintained below 0.5% v/v throughout the experiments.

### Cell Culture

The microglial BV-2 cell line was developed by Dr. E. Blasi from University of Perugia, Italy. The cells were cultured in Dulbecco’s Modified Eagle’s Medium (DMEM) containing of 10% (v/v) heat-inactivated fetal bovine serum (FBS), 100 unit/mL streptomycin/penicillin, and 50 μg/mL Amphotericin B. The cells were cultured in a 5% CO_2_ incubator at 37°C in a humidified atmosphere.

### Cell Viability Assay

The anti-neuroinflammatory effects of ESEAF against LPS-induced inflammation in BV-2 cells were evaluated by 3-(4,5-dimethylthiazol-2-yl)-2,5-diphenyltetrazolium bromide (MTT) assay. 1 × 10^4^ cells were seeded at each well in a 96-well plate and left to adhere for 24 h. Cells were pretreated with ESEAF (1.56–25 μg/mL) for 2 h and exposed to LPS (1 μg/mL) for 24 h. Subsequently, 5 mg/mL of MTT solution was added to each well for 4 h at 37°C. The absorbance was measured spectrophotometrically using microplate reader (Oasys UVM340) at 570 nm with reference wavelength of 650 nm (Supriady et al., 2015).

### DPPH Radical Scavenging Assay

DPPH free radical scavenging activity of ESEAF was measured according to the previous described method with minor modifications (Ser et al., 2015). ESEAF sample was diluted to a concentration in the range of 15.6–1000 μg/mL. For the test, 195 μL of 0.016% DPPH in 95% ethanol (Sigma) was added to 5 μL of sample solution. Gallic acid was used as positive control. Reaction mixture was incubated for 30 min at room temperature in darkness. The absorbance was measured at 515 nm using a spectrophotometer with methanol as the blank. The DPPH free radical scavenging activity was calculated as follows: DPPH scavenging activity (RSA) = (A_C_ - As)/A_0_ × 100%, where A_C_ is the absorbance of control; A_s_ is the absorbance of the sample.

### Superoxide Dismutase (SOD) Determination Assay

Superoxide dismutase scavenging activity of ESEAF was assessed according to the manufacturer’s method (19160 SOD determination kit-WST, Sigma–Aldrich). Twenty microliter of sample solution ranging from 0.488 to 31.25 μg/mL and mixture reaction solution were added to each well in a 96-well plate followed by incubation for 20 min at 37°C. The SOD activity of ESEAF was measured at 450 nm using a microplate reader. The SOD activity (percentage of inhibition %) was calculated as followed: SOD activity = (A_0_ - A_b_) - (A_s_ - A_sb_)/(A_0_ - A_b_) × 100%, where A_0_, Absorbance control blank; A_b_, Absorbance buffer blank; A_s_, Absorbance sample; A_sb_, Absorbance sample blank.

### Catalase Assay

Catalase activity was assessed according to the manufacturer’s instruction by using catalase assay kit from Sigma–Aldrich. After reaction with catalase, the colorimetric assay was measured based on the residual of hydrogen peroxide substrate by using a substituted phenol (3,5-dichloro-2-hydroxybenzenesulfonic acid), which oxidatively couples to 4-aminoantipyrine leading to the formation of N-(4-antipyryl)-3-chloro-5-sulfonatep-benzoquinone-monoimine. Ten microliter of catalase enzymatic reaction mixture added to 1 mL of color reagent and incubated for 15 min at room temperature. Catalase activity in each sample was measured at 520 nm and calculated as followed:

Activity (μmoles/min/mL) = (Δμmoles (H_2_O_2_) × dilution factor × 100)/(V × t), where V, sample volume; T, catalase reaction duration (minutes).

### Nitric Oxide Quantification Assay

The NO level was quantified by using Total Nitric Oxide Assay Kit (Thermo Fisher Scientific, Waltham, MA, United States) according to the manufacturer’s protocol. 10 × 10^3^ cells were seeded in a 96-well plate for 24 h followed by pretreatment with ESEAF (0.13–1.0 μg/mL) for 2 h prior to LPS exposure (1.0 μg/mL) for 4 h. Upon the completion of the incubation period, 50 μL of culture supernatant was mixed with 25 μL NADH and 25 μL of nitrate reductase and incubated for 30 min at 37°C. This was further followed by the addition of 50 μL of Griess reagents 1 and 2 for 10 min. Absorbance was measured and quantified by using Oasys UVM340 microplate reader at 570 nm. The concentration of NO was evaluated based on the nitrate standard curve.

### Measurement of Prostaglandin E2 (PGE_2_) Level

Microglia cells were cultured in 96-well plates, treated with different concentrations of ESEAF for 2 h prior to the stimulation of LPS for 24 h. The culture supernatant of each sample was collected to determine PGE_2_ level by using the Pierce^®^ PGE_2_ Competitive ELISA Kit (Thermo Fisher Scientific, Rockford, IL, United States).

### Determination of Intracellular Reactive Oxygen Species (ROS) Level

The fluorescent probe 2,7-dichlorodihydrofluorescein diacetate (DCFH-DA) was utilized to measure the generation of intracellular ROS level. 1 × 10^6^ cells were seeded in 60 mm^2^ culture dish and exposed to different concentrations of ESEAF ranging from 0.25, 0.5, and 1.0 μg/mL for 2 h followed by stimulation of LPS for 24 h. Cells were then stained with 10 μM DCFH-DA for 30 min. The fluorescence intensity of DCF was quantified by flow cytometer (BD Accuri C6) and detected in FL1-A channel (Goh et al., 2014).

### Western Blot Analysis

BV-2 cells (1 × 10^6^ cells) were seeded in 60 mm^2^ culture dishes and pretreated with different concentrations of 0.25, 0.5, and 1.0 μg/mL of ESEAF prior to the stimulation of LPS for 24 h. Then, the cells were harvested and lysed in cold RIPA buffer containing protease and phosphatase inhibitors. The total content of protein was quantified by using Bradford assay. Twenty-five microgram of total protein was separated by 10% or 12% SDS–PAGE and transferred onto a nitrocellulose membrane. Membrane was further blocked with skim milk or bovine serum albumin for 1 h and incubated with primary antibodies (COX-2, iNOS, NRF-2, HO-1, p65, p-p38 MAPK, p38 MAPK, lamin B and β-actin, Cell signaling; IL-1β, TNF-α, Thermo scientific) overnight at 4°C. Subsequently, the membrane was reacted with anti-rabbit/mouse immunoglobulin G-horseradish peroxidase-labeled secondary antibodies for 1 h at room temperature. The membrane was stained with enhanced chemiluminescence (ECL) detection kit and subjected to detection using gel documentation system. Band intensities were quantified by using Vilber Lourmat.

### Statistical Analysis

The data were analyzed by ANOVA followed by Dunnett’s test or Student’s *t*-test. *P* < 0.05 was considered statistically significant. All the data were expressed as means ± standard error (SE).

## Results

### Acute Toxicity, Behavior, Symptoms, and Mortality

There is no abnormality and no mortality in clinical signs, body weight and necropsy findings for all animals in the acute toxicity study after oral administration of ESEAF.

### Organ Weight/BW Coefficients

After 14 days, the body weight were recorded and the mice were sacrificed to collect organs. **Table 1** displayed the coefficients of various organs including liver, spleen, kidney, lung, and heart to body weight which are expressed as milligrams (wet weight tissues)/g (body weight). No apparent differences were observed in the body weight for both control and treatment groups. There is no significant differences were found in the coefficients of liver, spleen, kidney, heart, and lung.

**Table 1:** Organ weight/BW coefficients (Mean ± SE).

Groups	Body weight (g)		Liver	Kidney	Spleen	Lung	Heart
						
	Before	After					
Control	24.72 ± 0.86	27.94 ± 0.59	42.91 ± 1.56	13.58 ± 0.60	3.56 ± 0.07	8.54 ± 0.71	4.28 ± 0.68
Treated 2000 mg/kg	25.2 ± 0.75	26.88 ± 0.81	46.20 ± 0.77	12.69 ± 0.97	3.73 ± 0.11	11.88 ± 1.30	4.51 ± 0.82


### Histopathological Detection and Blood Biochemical Analysis

**FIGURE 1:**
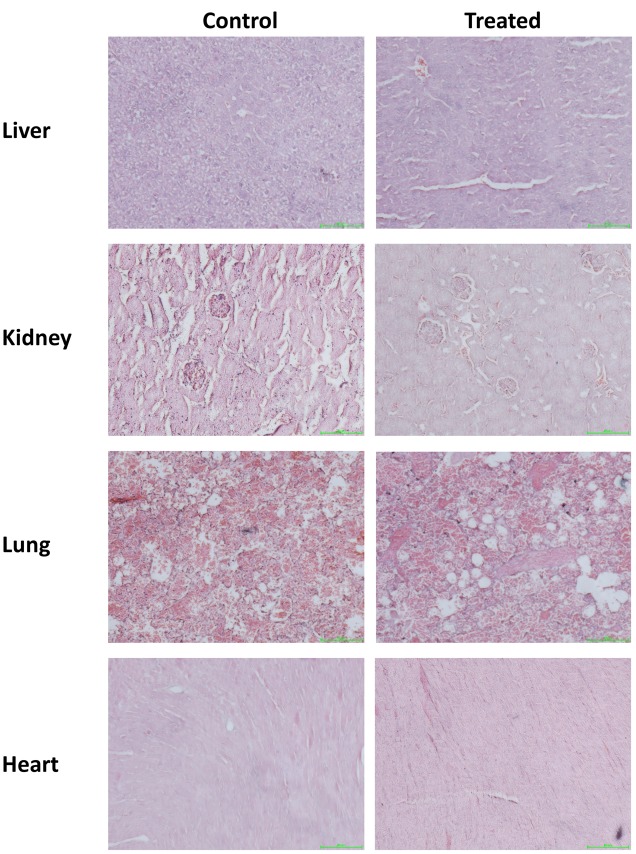
Histopathological examination of various organs including liver, heart, kidney, lung, and spleen when treated with 2000 mg/kg ethyl acetate fraction of *Elephantopus scaber* as compared to control. Magnification: 200×.

Gross examination during autopsy and histopathological lesions were not observed in any of the main organs which stained with H&E (**Figure 1**). Biochemical parameters in the serum detected by the autoanalyzer were listed in **Table 2**. No significant differences were discovered in the serum levels of CREA, albumin, TBIL, ALP, AST, and ALT in ESEAF treated mice as compared to the control group.

**Table 2:** Biochemical parameters in blood serum (mean ± SE).

Biochemical parameters	Control group	Treated group(2000 mg/kg)
Creatinine (CREA)	10.86 ± 0.40	9.83 ± 0.31
Albumin	25.71 ± 0.18	25.33 ± 0.67
Total bilirubin (TBIL)	0.71 ± 0.42	0.33 ± 0.21
Alanine phosphatase (ALP)	99.29 ± 5.02	97.00 ± 4.31
Aspartate aminotransferase (AST)	72.57 ± 12.15	111.50 ± 29.63
Alanine aminotransferase (ALT)	17.86 ± 2.35	23.83 ± 3.17


### Effects of ESEAF on the Cell Viability of BV-2 Cells against LPS-Induced Inflammation

**FIGURE 2:**
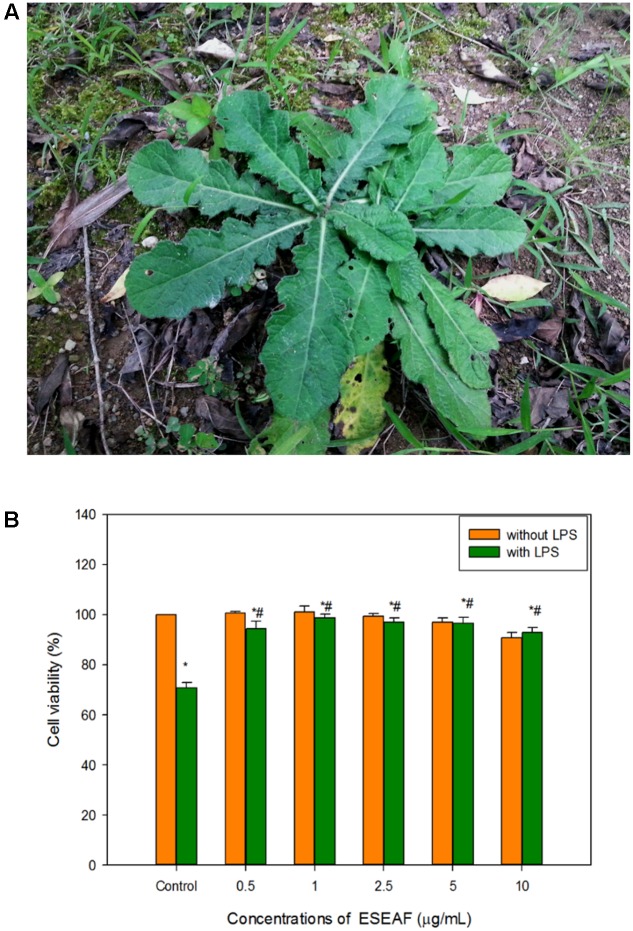
Cell viability of ESEAF on lipopolysaccharide (LPS)-induced BV-2 cells. **(A)** The image showed the *E. scaber*. **(B)** The bar chart indicated the percentage of cell viability of ESEAF in LPS-induced BV-2 microglia cells. Values are expressed in mean ± SE (*n* = 3). Asterisks indicate significantly different from the control groups (^∗^*p* < 0.05). Hashtags indicate significantly different from the LPS-treated group (^#^*p* < 0.05).

BV-2 cells were treated with ESEAF at different concentrations ranging from 0.5 to 10 μg/mL for 2 h with and without the presence of LPS for 24 h. Treatment with ESEAF only demonstrated that ESEAF did not induce obvious cytotoxic effect on the viability of BV-2 cells. Conversely, after treatment with LPS, results showed a significant decline in the cell viability with the value of 71.12 ± 0.73% as compared to the control. However, exposure to ESEAF (0.5–10 μg/mL) prior to the LPS treatment significantly enhanced the cell viability as compared to the treatment with LPS alone (**Figure 2B**) which further revealed the ability of ESEAF to mitigate the LPS-induced toxicity in BV-2 cells.

### Radical Scavenging Effect by ESEAF in DPPH Assay

**FIGURE 3:**
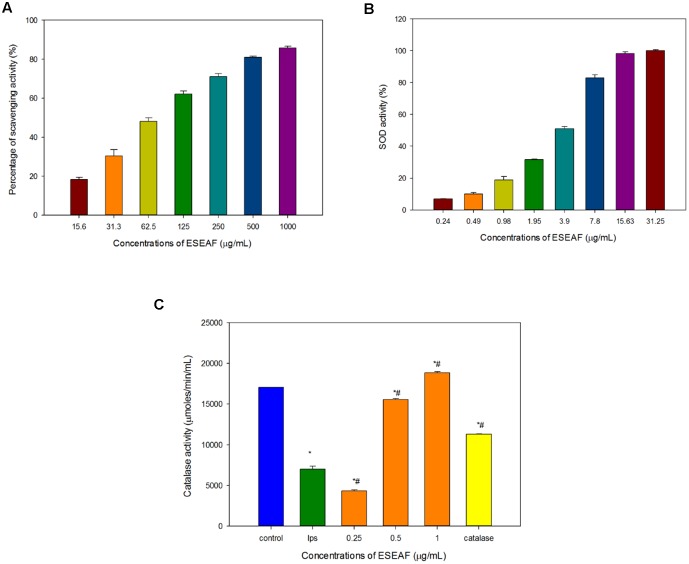
The antioxidant activities of ESEAF. **(A)** The bar chart displayed the percentage of scavenging activity for DPPH assay. **(B)** The bar chart indicated the superoxide dismutase (SOD) activity of ESEAF. **(C)** The bar chart indicated the catalase activity of ESEAF. Values are expressed in mean ± SE (*n* = 3). Asterisks indicate significantly different from the control groups (^∗^*p* < 0.05). Hashtags indicate significantly different from the LPS-treated group (^#^*p* < 0.05).

Antioxidant activity of ESEAF was assessed by determining the radical scavenging ability using DPPH assay. Reduction of the purple stable free radical DPPH by accepting an electron or hydrogen from antioxidant converts to a yellow colored diphenylpicrylhydrazine (Molyneux, 2004). The amount of free radicals present was measured by the decrease of the absorbance at 517 nm. ESEAF significantly reduced DPPH radicals as evident by the increase of radical scavenging activity with the increasing concentrations of ESEAF (15.6–1000 μg/mL) (**Figure 3A**). The highest inhibition of DPPH was demonstrated at 1000 μg/mL with the value of 85.73%. The IC_50_ value of ESEAF is 69.70 ± 0.01 μg/mL. Based on the results obtained from DPPH assay, it indicated that ESEAF possessed significant antioxidant effect.

### Effect of ESEAF on Superoxide Anion Radical Scavenging Activity

Superoxide radicals are dismutated by SOD, a major intracellular antioxidant enzyme, to form hydrogen peroxide and oxygen molecules (Scandalios, 1993). Thus, in this study, the ability of ESEAF to scavenge oxygen species such as superoxide anions were assessed. According to the results, the inhibition of superoxide anion radical generation gradually increased with the increasing concentration of ESEAF (**Figure 3B**). The results indicated that ESEAF appears as a potent superoxide anion scavenger with the significant IC_50_ value of 3.79 ± 0.16 μg/mL.

### Effect of ESEAF on Catalase Enzyme Activities

Catalase is one of the endogenous antioxidants that involved in cellular antioxidant defense (Day, 2009). As shown in **Figure 3C**, catalase activity was found to decrease when exposed to LPS alone. However, when pretreatment with different concentrations of ESEAF (0.25–1.0 μg/mL), catalase activity was remarkably elevated in a dose-dependent manner. ESEAF increased the catalase activity by 2.69-fold at the highest concentration of ESEAF as compared to the LPS alone treated group. This indicates ESEAF restores the antioxidant levels in LPS-stimulated BV-2 cells.

### ESEAF Suppressed NO and PGE_2_ Production in LPS-Stimulated BV-2 Cells

**FIGURE 4:**
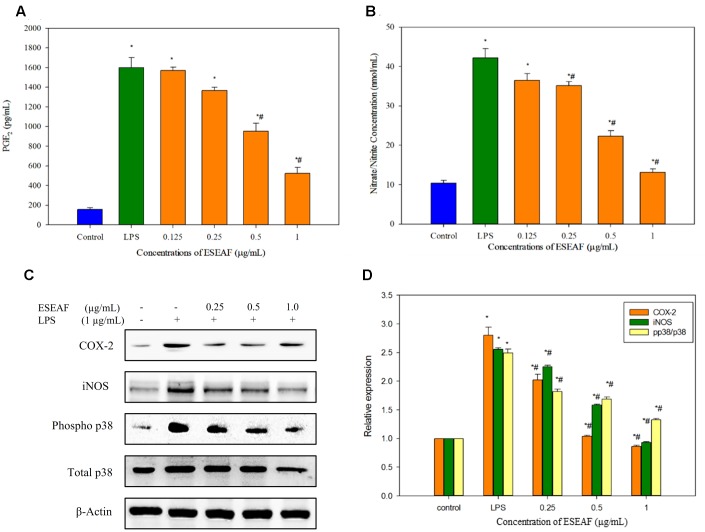
The anti-inflammatory effects of ESEAF in LPS-stimulated BV-2 cells. **(A)** The bar chart demonstrated the production of nitric oxide after pretreatment of ESEAF in LPS-stimulated BV-2 cells. **(B)** The bar chart demonstrated the production of prostaglandin E_2_ (PGE_2_) after pretreatment of ESEAF on LPS-stimulated BV-2 cells. **(C)** Western blot analysis on the inhibitory effect of ESEAF in LPS-induced iNOS, COX-2, p38, and phospho-p38 in BV-2 cells. β-actin was utilized as a loading control for all experiments. **(D)** The relative protein expression of iNOS, COX-2, and p-p38/p38. Asterisks indicate significantly different from the control groups (^∗^*p* < 0.05). Hashtags indicate significantly different from the LPS-treated group (^#^*p* < 0.05).

Excessive production of NO is one of the inflammatory hallmarks (Liu et al., 2002). To evaluate the effects of ESEAF on the pro-inflammatory mediator, production of NO was determined in LPS-induced BV-2 cells after pretreatment with different concentrations of ESEAF for 2 h. **Figure 4B** displayed that the NO production was significantly enhanced by 77.69% in LPS alone treatment group as compared to the control group. In contrast, pretreatment with increasing concentrations of ESEAF (0.125, 0.25, 0.5, and 1.0 μg/mL) significantly attenuated the LPS-induced NO production. The NO level was decreased to 36.54 ± 1.66, 35.173 ± 1.018, 22.28 ± 1.45, and 13.17 ± 0.87 nmol/mL at 0.125, 0.25, 0.5, and 1.0 μg/mL of ESEAF, respectively (**Figure 4B**). Subsequently, the effect of ESEAF on the production of PGE_2_ in LPS-stimulated BV-2 cells was examined by using PGE_2_ competitive ELISA kit. The production of PGE_2_ is an essential event in response to swelling and inflammation (Serhan and Savill, 2005). LPS alone significantly increased the production of PGE_2_ by 10-folds in BV-2 cells as compared to the untreated control group. As shown in the **Figure 4A**, ESEAF was demonstrated to inhibit the production of PGE_2_ in LPS-induced BV-2 cells.

### ESEAF Attenuated LPS-Induced iNOS and COX-2 Activation in BV-2 Cells

To further investigate the involvement of iNOS and COX-2 in the treatment of ESEAF against LPS-induced BV-2 cells, protein expression of iNOS and COX-2 were determined by using western blot analysis. Exposure of LPS in BV-2 cells markedly increased the iNOS protein expression whereas ESEAF suppressed the LPS-induced increase of iNOS protein expression in an increasing concentrations manner (**Figures 4C,D**). In addition, the results clearly showed that inducible COX-2 was prominently attenuated in a dose-dependent manner by ESEAF in LPS-induced BV-2 cells as compared to the LPS alone (**Figures 4C,D**).

### ESEAF Attenuated LPS-Induced p38 Phosphorylation

The effects of ESEAF on the activation of p38 phosphorylation in LPS-stimulated BV-2 cells was investigated. As shown in **Figures 4C,D**, the phosphorylation of p38 significantly promoted when exposed to LPS alone. However, when treatment with increasing concentrations of ESEAF, phosphorylation of p38 was significantly reduced in LPS-induced BV-2 cells when compared to the treatment of LPS alone.

### ESEAF Suppressed the LPS-Induced Translocation of NF-κB

**FIGURE 5:**
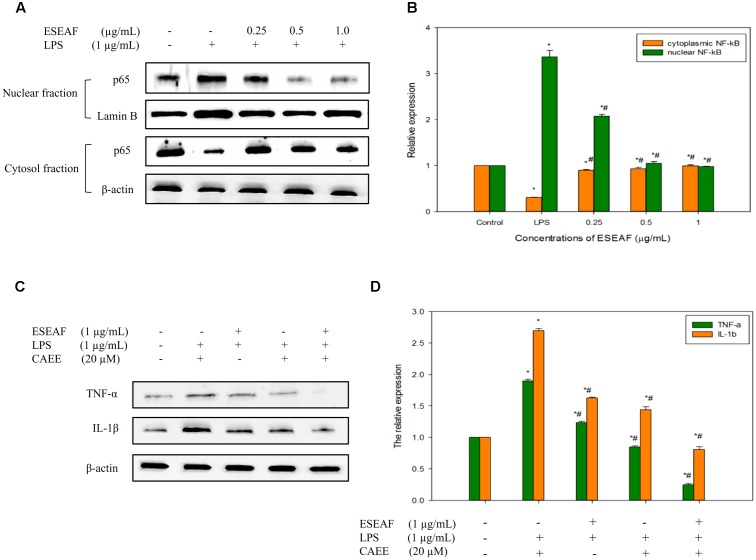
The regulatory effects of NF-κB on the pro-inflammatory cytokines by ESEAF in LPS-stimulated BV-2 cells. **(A)** The effect of ESEAF on LPS-induced translocation of p65 NF-κB. **(B)** The relative expression of the cytoplasmic and nuclear fractions of p65. **(C)** The anti-inflammatory effect of ESEAF on NF-κB-dependent pro-inflammatory cytokines in LPS-induced BV-2 cells. BV-2 cells were pretreated with CAEE (20 μM) prior to the exposure of ESEAF for 2 h and LPS for 24 h. **(D)** The relative expression of pro-inflammatory cytokines. β-actin was utilized as a loading control for all experiments. Values are expressed in mean ± SE (*n* = 3). Asterisks indicate significantly different from the control groups (^∗^*p* < 0.05). Hashtags indicate significantly different from the LPS-treated group (^#^*p* < 0.05).

Induction of inflammatory proteins including iNOS and COX-2 were stimulated by the NF-κB transcriptional factor (Tak and Firestein, 2001). To elucidate whether ESEAF suppressed the translocation of NF-κB to nucleus, protein expression of NF-κB subunit p65 was investigated by using western blot analysis. When exposed to LPS alone, p65 NF-κB subunit was found to translocate to the nucleus from the cytoplasm, evidenced by the increase of the p65 protein expression level (**Figures 5A,B**). However, pretreatment with ESEAF accumulated p65 protein in the cytosol and accompanied by the decrease of p65 protein in nuclear fraction indicating inhibition of LPS-induced translocation of p65.

### ESEAF Modulated NF-κB-Dependent Pro-inflammatory Cytokines

Activation of NF-κB results in the release of pro-inflammatory cytokines such as IL-1β and TNF-α which act as triggers of the neuroinflammation onset (McCoy and Tansey, 2008; Glass et al., 2010). In this study, the effect of ESEAF on the production of cytokines (TNF-α and IL-1β) was investigated in LPS-stimulated BV-2 cells. Upon stimulation with LPS, the production of pro-inflammatory cytokines, TNF-α and IL-1β was significantly elevated as compared to the untreated group (**Figures 5C,D**). However, LPS-induced pro-inflammatory cytokines generation was reversed by the pretreatment with ESEAF. These findings indicated that ESEAF ameliorated the inflammatory response by suppressing the release of pro-inflammatory cytokines. To further substantiate the effect of ESEAF on the correlation between the regulation of NF-κB and the cytokines production, BV-2 cells were exposed to ethyl 3,4-dihydroxycinnamate (CAEE), a NF-κB inhibitor prior to the LPS stimulation. It was observed that CAEE significantly reduced the LPS-induced TNF-α and IL-1β as compared to the LPS- and ESEAF-treated groups. Furthermore, ESEAF was found to further decrease the level of pro-inflammatory cytokines TNF-α and IL-1β, demonstrating that ESEAF potentiated the anti-neuroinflammatory effect upon pretreatment with CAEE. Thus, this data indicated that ESEAF modulated pro-inflammatory cytokines via the inactivation of NF-κB in LPS-stimulated BV-2 cells.

### Effect of ESEAF on the Intracellular Reactive Oxygen (ROS)

**FIGURE 6:**
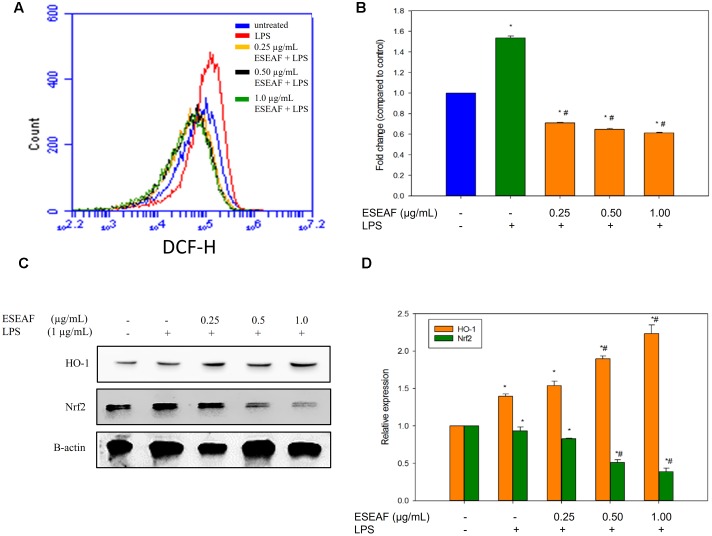
Attenuation of oxidative stress via activation of Nrf2 and heme-oxygenase-1 (HO-1) by ESEAF in LPS-induced BV-2 cells. **(A)** The histogram showed the intracellular reactive oxygen species (ROS) level after pretreatment with ESEAF. **(B)** The bar chart summarized the fold change of the intracellular ROS level in LPS-induced BV-2 cells. **(C)** Western blot analysis on the activation of Nrf2 and HO-1 protein by ESEAF in LPS-induced BV-2 cells. β-actin was utilized as a loading control for all experiments. **(D)** The bar chart showed the relative protein expression of Nrf2 and HO-1 when treat with different concentrations of ESEAF (0.25–1.0 μg/mL). Values are expressed in mean ± SE (*n* = 3). Asterisks indicate significantly different from the control groups (^∗^*p* < 0.05). Hashtags indicate significantly different from the LPS-treated group (^#^*p* < 0.05).

The intracellular ROS level was measured by using a fluorescent probe, DCFH-DA dye. Treatment with LPS alone instigated a significant accumulation of intracellular ROS level in BV-2 cells as compared to the untreated cells which evident by the right shift in the histogram (**Figure 6A**). On contrary, exposure of ESEAF prior to the stimulation of LPS abrogated the increase of LPS-induced ROS generation. As shown in **Figure 6B**, ROS level was reduced by 0.61 ± 0.01-fold at 1.0 μg/mL of ESEAF in LPS-stimulated BV-2 cells.

### ESEAF Induced Activation of Nrf2 and HO-1 Protein Expression

It was reported that the activation of Nrf2/HO-1 pathway attenuates oxidative stress and inflammation in neurodegenerative diseases (van Muiswinkel and Kuiperij, 2005; Paine et al., 2010). To investigate the involvement of Nrf2 and HO-1 proteins, ESEAF was exposed to LPS-stimulated BV-2 microglia cells. In our findings, the protein expression of Nrf2 in cytoplasmic fractions was detected to gradually decrease when treated with ESEAF in LPS-induced BV-2 cells when compared to the LPS treatment alone, indicating the Nrf2 translocate to nucleus (**Figures 6C,D**). Next, HO-1 is one of the downstream mediators of Nrf2 pathway. Hence, the expression level of HO-1 was assessed to evaluate whether it exerts its protection roles in the present study. As shown in **Figure 6C**, treatment of ESEAF resulted a dose-dependently upregulation of HO-1 protein expression in BV-2 cells. This suggested that ESEAF activates Nrf2 via HO-1 induction in LPS-stimulated BV-2 cells.

## Discussion

Activation of microglia cells promotes the aberrant formation of inflammatory mediators such as NO and PGE_2_. This is regarded as a characteristic of neuroinflammation which contributes to the damage in neurons and subsequently leading to neurodegenerative disorders (Block et al., 2007). Growing evidence also supports the role of ROS in regulating the production of NO and PGE_2_ and results in inflammatory responses in microglia (Bauer and Bauer, 2002; Jayasooriya et al., 2015; Jeong et al., 2015). In the present study, ESEAF was found to mitigate the production of NO and PGE_2_ in LPS-induced BV-2 cells. The neurotoxic NO is generated from L-arginine catalyzed by inducible isoform of NO synthase (iNOS). Abnormal increase of iNOS level speeds up the elevation of NO production which is considered to act as a potent neurotoxin that damages lipids, DNA and proteins (Bloodsworth et al., 2000; Aktan, 2004). However, ESEAF found to inhibit iNOS expression in LPS-induced BV-2 cells. It is well documented that the successive conversion of PGE_2_ from arachidonic acid which is aided by the high level of COX-2 enzymes exacerbates inflammatory response and leading to the development of neurological disorders (Simon, 1999; Minghetti, 2004). Hence, it is conceivable that down-modulation of PGE_2_ production concomitantly with the inhibition of COX-2 in the brain is a potent means of anti-neuroinflammatory targeted approach. In connection with this, the current findings demonstrated ESEAF exerting its anti-inflammatory effect through attenuation of LPS-induced COX-2 protein expression in BV-2 cells.

The activation of NF-κB transcription factor triggers the onset of inflammation in microglia in response to the oxidative stress (Mattson and Camandola, 2001). Previous evidence showed that the translocation of NF-κB to nucleus promotes the activation of pro-inflammatory enzymes including iNOS, COX-2 resulting in pathogenesis of neurodegenerative diseases (Baeuerle and Baltimore, 1996; Mattson and Camandola, 2001). Inhibition of NF-κB transcriptional activity in microglia may modulate the progression of neurodegenerative diseases by reversing the neuronal death which has become a molecular target of interest for the discovery and development of anti-inflammatory therapeutic drugs. Additionally, activation of NF-κB is reported to closely associate with MAPK signaling transduction including p38, JNK, and ERK which contributes to the initiation of neuroinflammation in response to several stimuli (Kaminska, 2005; Jung et al., 2007). Notably, p38 MAPK, an important upstream modulator, predominantly stimulated by LPS resulting in NF-κB translocation and further activation of iNOS and COX-2 in microglia (Kim et al., 2006). Thus, most of the anti-inflammatory drugs were found to modulate iNOS and COX-2 via inhibit NF-κB and p38 signaling. For instance, resveratrol and curcumenol, natural plant derivatives, suppressed iNOS and COX-2 via inhibition of NF-κB and p38 signaling pathways (Zhong et al., 2012; Lo et al., 2015). This is concordant with our current findings which demonstrated that treatment with ESEAF suppressed the phosphorylation of p38 by attenuating the translocation of NF-κB along with the alteration of iNOS and COX-2 upon stimulation of LPS in microglia cells, suggesting that ESEAF is a potent inhibitor of p38.

Upon prolonged microglial activation, constitutive activation of NF-κB leads to dysregulation of cytokines which contributes to the pathogenesis of neurodegenerative diseases particularly in AD via amplification of β-amyloid (Aβ) synthesis (Chen et al., 2012; Wang et al., 2015). Therefore, targeting the pro-inflammatory cytokines such as TNF-α and IL-1β, is considered as one of the most promising therapeutic approaches to alleviate inflammation and prevent the accumulation of Aβ in the brain (Heneka et al., 2015). In current scenario, ESEAF was shown to effectively ameliorate the inflammatory response by suppressing the production of TNF-α and IL-1β. These findings are in accordance production with the recent reports that celastrol and resveratrol attenuated the microglial activation via diminishing TNF-α and IL-1β level (Jung et al., 2007; Zhong et al., 2012). Interestingly, pretreatment of ESEAF abrogated the LPS-induced secretion of TNF-α and IL-1β through the inhibition of NF-κB. The finding was further substantiated by the use of CAEE, a specific NF-κB inhibitor, which is in parallel with the observation in ESEAF-treated group.

Oxidative stress occurs when the excessive ROS is generated concomitantly along with the dysfunction of endogenous antioxidant system (Barnham et al., 2004). In CNS, accumulation of ROS levels triggers activation of microglia and further exacerbates neuroinflammation responses leading to the progression of neurodegenerative disorders (Uttara et al., 2009; Hsieh and Yang, 2013). Mechanistically, aberrant production of ROS provoked inflammatory responses which aberrantly unleash a variety of pro-inflammatory factors such as NO and PGE_2_ in microglia via activation of diverse downstream signaling mediators including NF-κB and MAPK (Rosenberger et al., 2001; Park et al., 2015). As a result, attenuation of ROS generation in microglia cells becomes a common therapeutic target for neuroinflammation-related diseases. Consistent with this notion, our results revealed that ESEAF efficiently reversed the production of ROS in LPS-induced BV-2 cells when compared to the LPS alone which caused a significant accumulation of ROS. These data indicated that ESEAF restores redox balance and protects microglia through scavenging of the intracellular ROS.

Mounting scientific evidences have reported that treatment with anti-inflammatory agents which possess antioxidant effect may attenuate the activation of microglia and thus promote neuronal cell survival in various neurodegenerative disease (Block and Hong, 2005; Smith et al., 2012). Thus, the antioxidant effect of *E. scaber* was investigated in the present study. Antioxidants are responsible in removing free radicals, scavenging ROS or preventing the excessive generation of ROS which mitigate the inflammatory and carcinogenic responses (Valko et al., 2006). DPPH assay is a common evaluation assay for antioxidant which based on the reduction of DPPH free radical by accepting hydrogen atom or electron resulting in de-colorization (Molyneux, 2004). A significant DPPH scavenging activity was exhibited by ESEAF which suggests the presence of potent antioxidants in the extract. Activated microglia increases proliferation and produces various cytotoxic factors including NO, superoxide radicals (O_2_^-^), and ROS (Colton and Wilcock, 2010; Ha et al., 2012) which results in various neurodegenerative diseases. Thus, enzymatic antioxidants including SOD, catalase, and glutathione peroxidase play important roles in the detoxification of the deleterious free radicals to protect cells from oxidative stress (Limón-Pacheco and Gonsebatt, 2009). Particularly, SOD enzymes catalyze the conversion of superoxide radical (O_2_^-^) into hydrogen peroxide (H_2_O_2_) and oxygen (Weydert and Cullen, 2010) whereas catalase converts H_2_O_2_ into water and oxygen. Our findings showed that the catalase activity was dose-dependently increased by ESEAF. Additionally, ESEAF also exhibited strong scavenging activity on superoxide radicals evident by its IC_50_ value of 3.79 ± 0.16 μg/mL. These collective evidences further strengthen the antioxidant potential of ESEAF.

**FIGURE 7:**
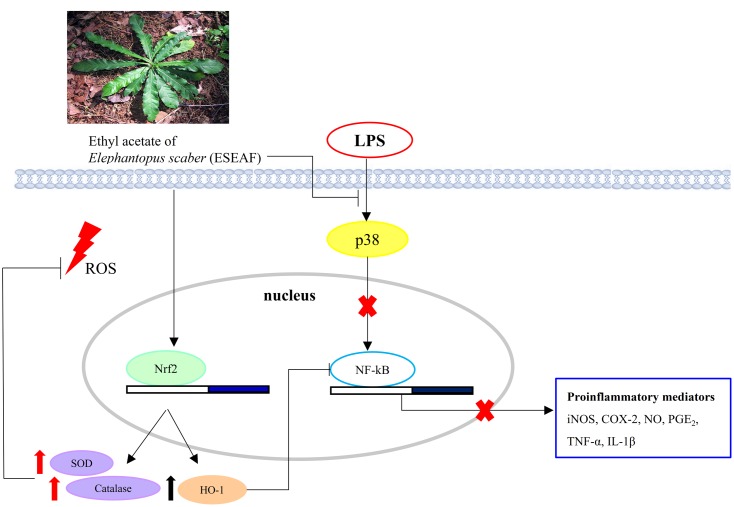
Schematic illustration depicting the potential pathways associated with the anti-inflammatory effects of ESEAF on LPS-stimulated BV-2 microglia cells.

Nrf2 which is recognized as a chief regulator of the antioxidant response is activated in response to oxidative stress (Hybertson et al., 2011). Conversely, during the inflammation conditions, the dysregulation of Nrf2 pathway and antioxidant defense system are found in neuronal and brain injury. In spite of this, accumulating evidences revealed that activation of Nrf2 signaling by plant derived therapeutic agents induces antioxidative response against inflammation-related chronic diseases and ameliorates oxidative stress via modulation of a multitude of genes including HO-1, SOD, and catalase (van Muiswinkel and Kuiperij, 2005; Gonzalez-Reyes et al., 2013; Lee et al., 2013). This is in line with our current findings showing the nuclear translocation of Nrf2 upon exposure to ESEAF in LPS-induced BV-2 cells enhanced the levels of antioxidant enzyme SOD and catalase along with the reduction of ROS level. Additionally, it is noteworthy that crosstalk between HO-1 and NF-κB, which is predominantly governed by Nrf2 plays important roles in targeting inflammation (Surh and Na, 2008; Paine et al., 2010). In response to oxidative stress, activated Nrf2 induces activation of ARE encoding HO-1 gene further represses NF-κB and modulates the inflammatory mediators, iNOS and COX-2 (Srisook and Cha, 2005; Wakabayashi et al., 2010). Therefore, activation of Nrf2/HO-1 pathway in microglia emerges as target of interest for drug discovery to prevent microgliosis (Foresti et al., 2013; Kang et al., 2013). In this regard, quercetin and 3,4,5-trihydroxycinnamic acid were reported to attenuate inflammatory response via Nrf2/HO-1 pathway by inhibiting NF-κB in LPS-induced BV-2 cells (Lee et al., 2014; Sun et al., 2015). Similarly, ESEAF was found to activate the Nrf2/HO-1 signaling and repress NF-κB followed by the modulation of downstream pro-inflammatory mediators in LPS-induced BV-2 cells. Collectively, ESEAF functions as an anti-inflammatory agent which may attribute to the presence of terpenoids including isodeoxyelephantopin, deoxyelephantopin, and lupeol that is known to possess anti-inflammatory effect (Bani et al., 2006; Ichikawa et al., 2006; Vasconcelos et al., 2008; Huang et al., 2010). This is substantiated by the GC-MS phytochemical profiling which is reported in our previous study whereby similar phytochemicals were detected in ESEAF (Chan et al., 2015). Thus, the presence of these constituents in ESEAF may be accountable for the observed anti-inflammatory effects in LPS-induced BV-2 cells. Taken together, the signaling pathways mediated by ESEAF in LPS-stimulated BV-2 cells were summarized and depicted in **Figure 7**.

Acute oral toxicity study is used to determine the safety and risk assessment of *E. scaber* in human health. Our present findings demonstrated that the mice which were orally administrated with 2000 mg/kg of ESEAF showed no overt signs of distress and no any toxic symptoms or deaths after 14 days treatment. This was further supported by the results showing no changes in organ coefficient and no structural changes in histopathological analysis of heart, kidney, spleen, lungs, and liver. Moreover, the overall body weight was found normal and exhibited a LD_50_ value of greater than 2000 mg/kg which reckoned that ESEAF is considered to be safe.

## Conclusion

In summary, the present study was the first to provide new insight on the acute toxicity, antioxidant and anti-inflammatory activities of ethyl acetate fraction of the leaves from *E. scaber*. Our findings revealed that ESEAF abrogated the exacerbation of LPS-induced iNOS, COX-2, IL-1β, and TNF-α via attenuation of the p38 and NF-κB. In addition, it is noteworthy that ESEAF ameliorated the oxidative stress induced by LPS through the activation of Nrf2 and HO-1 to boost the scavenging activity of antioxidants as well as to suppress the inflammatory response via NF-κB signaling in BV-2 cells. In addition, ESEAF is suggested to be safe for therapeutic use with no toxic symptoms observed at 2000 mg/kg based on the acute toxicity test. In short, our collective results provide scientific evidence to support the ethnomedicinal use of *E. scaber* in alleviating the inflammatory response in neurodegenerative diseases. Therefore, the current findings have further substantiated the potential of ESEAF to be developed as a potential therapeutic agent for the treatment of microglial-mediated neuroinflammatory diseases.

## Author Contributions

The experiments, data analysis and manuscript writing were conducted by C-KC, SA, MK, and LT. B-HG and HK contributed by providing vital technical support for the project and proofread on the writing. HK contributed to the funding of the project. B-HG and HK founded the research project.

## Conflict of Interest Statement

The authors declare that the research was conducted in the absence of any commercial or financial relationships that could be construed as a potential conflict of interest.
